# Epigenetic Changes Associated With a Phenotypic Transition in Namibian Cheetahs

**DOI:** 10.1111/mec.70527

**Published:** 2026-09-07

**Authors:** Colin Vullioud, Diana Geweiler, Joerg Melzheimer, Sonja Heinrich, Jörns Fickel, Bettina Wachter, Alexandra Weyrich

**Affiliations:** ^1^ Department of Evolutionary Genetics Leibniz‐Institute for Zoo and Wildlife Research (IZW) Berlin Germany; ^2^ Chair of Modelling of Social‐Ecological Systems University of Freiburg Freiburg im Breisgau Germany; ^3^ University of Potsdam Potsdam Germany; ^4^ Department of Evolutionary Ecology Leibniz‐Institute for Zoo and Wildlife Research (IZW) Berlin Germany

**Keywords:** conservation biomarker, DNA methylation, life‐history transition, low genetic diversity, spatial tactic, threatened species

## Abstract

In populations with low genetic diversity, the capacity to adapt to environmental change is limited. While epigenetic mechanisms can generate phenotypic plasticity, their role in mediating adaptive life‐history transitions in the wild is largely unknown. Here, we show that in adult Namibian cheetahs ( *Acinonyx jubatus jubatus* ), a species with remarkably low genetic diversity, the transition from a floater to a male holding a territory is associated with widespread DNA methylation changes, with a predominance of hypermethylation in territorial males. Using paired blood samples collected from the same individuals before and after becoming territorial, we identified differentially methylated genes which function in pathways related to anatomical and head development, brain development and neurodevelopment, cAMP biosynthesis and ion channel transport, consistent with the observed phenotypic shifts in morphology, spatial movement and aggressive behaviour. These findings suggest that DNA methylation mediates this life‐history transition at the molecular level. More broadly, they provide rare evidence that epigenetic regulation is relevant in phenotypic diversity within species with low genetic diversity, potentially supporting adaptive potential at the population level.

## 1 Introduction

The current extinction crisis is marked by a global decline in biodiversity, with losses at the levels of species, populations and genetic variation. As populations shrink, inbreeding intensifies and genetic variation decreases, driving a self‐reinforcing decline towards extinction known as the extinction vortex (Bosse and van Loon 2022).

While reduced genetic diversity limits the adaptive potential of species and increases their vulnerability to environmental change (Shaw et al. 2025), field observations suggest that some species with low genetic variability remain resilient (Pečnerová et al. 2021). This suggests that other mechanisms, beyond genetic variation, increase species adaptability. Epigenetic modification is one candidate mechanism as it can regulate gene expression without changing the DNA sequence and contribute to the development of multiple traits (Bird 2002; Szyf 2009; Szyf and Bick 2013). DNA methylation patterns mediate both long‐term developmental programming and dynamic short‐term responses to environmental cues (Jablonka and Raz 2009; Szyf 2009, 2011). While epigenetic studies have largely focused on responses to external drivers such as temperature (Ryu et al. 2020; Weyrich et al. 2016, 2020), diet (Lea et al. 2016; Weyrich et al. 2018), social conditions (Guerrero et al. 2020; Vullioud et al. 2024), captivity (Weyrich et al. 2022) or pollution (Beck et al. 2022; Skinner 2007), its role in intrinsic phenotypic transitions, including morphological changes, has remained mostly unexplored.

These transitions, rare in nature, represent natural experiments that allow us to understand how a single genotype can give rise to multiple phenotypes at the individual level and to disentangle genetic from epigenetic effects (Abdolmaleky et al. 2025; Stearns 1989; West‐Eberhard 1989). Other regulatory mechanisms, such as transcription factor activity, complement gene expression regulation (Martin 2007; Volanakis 1995). While life‐history transitions in single individuals are not adaptation in itself, at the population level, such individual plasticity expands the range of phenotypic variations, which can enhance the population's capacity to respond to environmental pressures, even when genetic diversity is low.

Here, we investigate the epigenetic changes of non‐territorial Namibian male cheetahs ( *Acinonyx jubatus jubatus* ), that is, ‘floaters’ that become territorial. All fully‐grown, sexually mature males start their adulthood as floaters. While being a solitary species, both floaters and territorial males often group in coalitions of two to four relatives (Caro 1994). When the coalition size of floaters is larger or the same size as that of territorial males, floaters might succeed to take over a territory after a deathly fight (Caro 1994; Melzheimer et al. 2018) to become territorial, while the others remain floaters. The transition from floater to territorial males is followed by significant trait changes, a rare phenomenon in mammals: territorial males gain muscle mass, increase head size, and display increased aggression and boldness, including towards humans (Melzheimer et al. 2018), thus undergoing a phenotypic transition from wide‐ranging floaters to small‐ranging territorial males (Melzheimer et al. 2018, 2020). Namibian cheetah are a resilient species, with a low genetic diversity, and two male phenotypes that change within a lifespan, representing a valuable candidate species to study epigenetic modifications in a wild mammal (Castro‐Prieto et al. 2011; Heinrich et al. 2017; Thalwitzer et al. 2010).

Here, we analysed DNA methylation patterns of peripheral blood mononuclear cells (PBMCs) collected during a long‐term study of Namibian cheetahs. We sampled 10 males twice, first as floater and later as territorial male, leading to a powerful paired‐sample design to disentangle epigenetic changes from the confounding influence of genetic variation. Our study had three goals: (i) to identify differentially methylated regions (DMRs) associated with the phenotypic transition; (ii) to determine whether these DMRs were located in genes and involved in biological processes consistent with the observed shifts in morphology and behaviour, while accounting for age; and (iii) to evaluate whether these epigenetic signatures could serve as predictive biomarkers to distinguish between floater and territorial spatial tactics. Since males are always older when they are territorial than when they are floaters, age is a potential confounding factor of any methylation difference between the two spatial tactics, which we took into account in our analyses.

## 2 Material and Methods

### 2.1 Study Animals and Sample Collection

Within the long‐term Leibniz‐IZW Cheetah Research Project in Namibia, free‐ranging male cheetahs were captured in box traps at cheetah marking sites (Melzheimer et al. 2020). Immobilization, medical check‐ups, weighting and biological sample collection followed established protocols (Thalwitzer et al. 2010). Adult cheetahs were fitted with GPS collars to monitor spatial movements and to determine their spatial tactic as floaters or territorial males (Melzheimer et al. 2018, 2020). For this study, we used paired blood samples from 10 clinically healthy adult males, each male was sampled first as a floater and later was recaptured and sampled again as a territorial male (Table S1). We assessed the age of the animals following Caro (1994) using the wear and tear of teeth, number of scars and shape of fur and allocated the ages into three age classes. Age class 6: > 2.0 to ≤ 3.5 years, age class 7: > 3.5 to ≤ 7.0 years and age class 8: > 7.0 years (Caro 1994). The time elapsed between the two sampling events was 25.7 ± 16.7 months (mean ± SD; range: 6.2–59.9 months). We made use of body mass measures of these 10 males (ID1‐ID10, Table S1) and of additional eight floaters that remained floaters (ID11‐ID18, Table S1).

### 2.2 PBMC Isolation

Whole blood was collected in heparin or citrate vacutainers, and ETDA vacutainers. PBMCs were isolated from heparin or citrate vacutainers within 24 h by density gradient centrifugation using Biocoll separating solution (Biochrom GmbH). Isolated PBMCs were washed, resuspended in freezing medium (90% foetal bovine serum, 10% DMSO) and cryopreserved at −80°C in a MrFrosty container for a few hours before transferred into liquid nitrogen until and during transport. After arrival at the Leibniz‐IZW, the samples were stored at −80°C. Blood smears were made in duplicates from EDTA vacutainers and differential cell blood counts performed by microscopy. Because isolated PBMCs consist of only lymphocytes (70%–90%) and monocytes (10%–30%), we counted the percentage of lymphocytes in the blood smears as a measure for infection. The percentages of lymphocytes in the blood smears in floaters and territorial males was similar and relatively low, thus indicating no acute infection in cheetahs when sampled (10 floaters: mean = 13.30% ± 7.70% becoming territorial males: mean = 12.50% ± 5.08% [mean ± SD]; eight floaters in first capture: mean = 11.57% ± 10.18% [no data for one ID] that had remained floaters in recapture: mean = 9.00% ± 4.56% [no data for two IDs]; Table S1).

### 2.3 DNA Extraction, Enrichment of Methylated DNA, Library Preparation and MBD‐Seq

Genomic DNA was extracted from PBMCs using the QIAamp DNA Blood Mini Kit (Qiagen, Hilden, Germany). DNA was sheared to 300–400 bp fragments using a Covaris M220 sonicator. Methylated DNA was enriched using the MethylMiner Methylated DNA Enrichment Kit (Thermo Fisher Scientific, Waltham, Massachusetts, USA), which employs biotinylated methyl‐binding‐domain protein 2 (MBD2) to capture methylated DNA fragments. Libraries for MBD‐Seq were prepared (Fortes and Paijmans 2015), involving end‐repair, dA‐tailing, Illumina‐specific adapter ligation and indexing PCR with one barcode per sample. Library quality and fragment size (200–700 bp) were assessed using a TapeStation 2200 (Agilent, Santa Clara, California, USA). All 20 barcoded libraries were pooled and sequenced on a single lane of an Illumina NovaSeq 6000 (S1 flow cell, 300 cycles, paired‐end) at the Max Delbrück Centre (MDC Berlin, Germany) to minimize technical batch effects.

### 2.4 Bioinformatics Processing

We processed the raw sequencing reads into clean reads: we removed adapters using cutadapt v2.4 (Martin 2011), and filtered reads for a Phred quality score ≥ 33 using Trimmomatic (v0.39). We mapped clean reads to the cheetah reference genome (VMU_Ajub_asm_v1.0, GCF_027475565.1) using Bowtie 2 v2.5.4 (Langmead and Salzberg 2012) in paired‐end mode (‐1, ‐2) using 10 parallel threads (‐p 10), end‐to‐end alignment mode (‐‐end‐to‐end), and excluded unaligned reads from the output (‐‐no‐unal). Alignment statistics were captured for each sample via stderr logging. Output was written in SAM format (‐S). We used SAMtools v1.21 (Danecek et al. 2021) to convert SAM to BAM format (‐b), retaining only reads with a minimum mapping quality score of 30 (‐q 30), including the header in the output (‐h), using 2 threads (‐@ 2). We segmented the reference genome into non‐overlapping 300 bp windows. Read counts per window were computed for all samples using HTSeq‐count v2.0 (Putri et al. 2022) applied to position‐sorted BAM files in unstranded mode (‐‐stranded no), with a minimum mapping quality of 20 (‐‐minaqual 20), intersection‐nonempty counting mode (‐‐mode intersection‐nonempty), and random assignment of reads overlapping multiple windows (‐‐nonunique random). Each 300 bp window represents a unit of analysis. We obtained genome annotation (GCF_027475565.1‐RS_2023_04) from NCBI, and added transcription start sites (TSS) as the first position of the first exon and the promoter regions as 2 kb upstream the TSS. We included this extended annotation file into the differential methylation analysis.

### 2.5 Differential Methylation Analysis Within a Multi‐Method Cross‐Validation Framework

**FIGURE 1:**
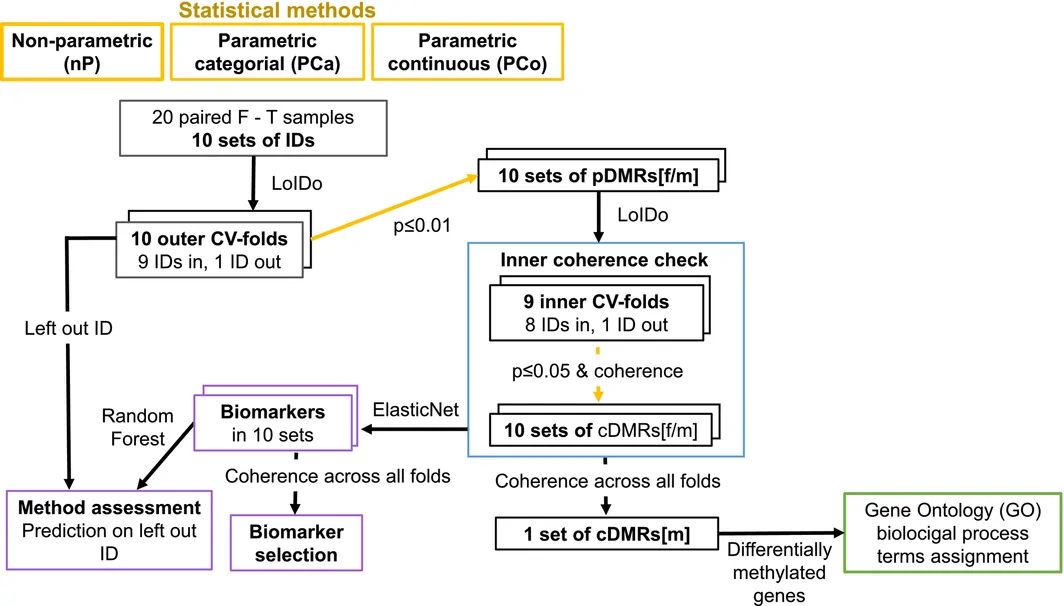
Analytical framework workflow. The analysis begins with the full dataset of 20 paired samples from 10 individuals (IDs) first as floaters (F) and then as territorial males (T). A leave‐one‐ID‐out (LoIDo) cross‐validation (CV) creates 10 ‘outer CV‐folds’, each with a training set (9 IDs) and a test set (the 1 left‐out ID, as unseen data). The multiple data sets are indicated by double rectangles. Within each training set, three parallel statistical methods (yellow boxes) are applied (yellow arrows): a non‐parametric test (nP), a parametric categorical analysis (PCa), and a parametric continuous analysis (PCo). Regions with *p* ≤ 0.01 are considered potential DMRs (pDMRs[*f*/*m*], *m* refers to method‐specific, and *f* to fold‐specific) and are subjected to an ‘inner coherence check’ (blue box). This involves a nested Leave‐one‐ID‐out‐cross‐validation (LoIDo‐CV), creating nine ‘inner CV‐folds’ (8 IDs in, 1 ID out). Each pDMR[*f*/*m*] is confirmed as a fold‐specific, method‐specific coherent DMR (cDMRs[*f*/*m*], *c* refers to coherent) if it remains significant (*p* ≤ 0.05) with a consistent direction of methylation change across all nine inner folds. Method‐specific cDMRs (cDMRs[*m*]) are the cDMRs[*f*/*m*] coherent across all 10 outer folds. We used cDMRs[*m*] overlapping genes for downstream gene ontology (GO) term assignment. For biomarker selection, an ElasticNet model was applied to the cDMRs[*f*/*m*] within each outer fold's training set. A Random Forest model was then trained on these selected biomarkers and its predictive accuracy was assessed on the corresponding left‐out ID (as unseen data). The final set of potential biomarkers is the set of biomarkers coherent across folds.

To detect methylation differences associated with the spatial tactic‐shift despite the small sample size, we combined a nested cross‐validation (CV) design, three complementary statistical methods and a coherence‐based selection of DMRs into a single analytical framework (Figure 1). Here, we describe in turn the CV structure, the three methods, DMRs identification and biomarkers selection.

We created a nested ‘Leave one ID out—Cross Validation’ (LoIDo‐CV) data structure (detailed in Supporting Information ‘General statistical approach’, Figure 1) as follows: We split the full dataset into 10 outer CV‐folds, each consisting of nine IDs in and one ID out. The nine individuals (18 samples per fold) constituted the training set and the left‐out ID the test set used for the predictive power assessment. We further split each of these outer CV‐folds into nine nested sub‐folds, using the same LoIDo approach; that is, an additional individual—from the nine in the outer CV‐fold's training set—was left out. This resulted in 10 outer differential methylation analysis on read counts per 300 bp window on nine individuals (18 samples) and 90 (10 outer folds × 9 inner sub‐folds) nested inner analyses, each performed on eight individuals (16 samples).

We identified DMRs between floaters and territorial males (‘tactic’) using the following methods:

1. Non‐parametric categorical (nP): A paired Wilcoxon signed‐rank test between the normalized read counts (using edgeR's calcNormFactors and cpm functions; Trimmed Mean of M‐values [TMM]) of the floater and territorial sample of each individual.
2. Parametric categorical (PCa): A negative binomial generalized linear model (GLM) fitted using edgeR (Robinson et al. 2010) and the following design matrix: ~ tactic + ID, where ‘tactic’ is a binary factor (floater/territorial) and ‘ID’ accounts for individual pairing. Dispersion was estimated using the estimateDisp function (empirical Bayes shrinkage for analysis on the outer folds; tagwise dispersion for analysis on the inner folds due to smaller sample size than the outer fold and analysis on a subset of windows). Differential methylation was assessed using a quasi‐likelihood *F*‐test (glmQLFit + QLFTest) for outer folds and a likelihood ratio test (glmFit + glmLRT) for inner folds.
3. Parametric continuous (PCo) analysis: A negative binomial GLM fitted using edgeR, modelling read counts as read_count ~ body_mass + ID, where ‘body_mass’ was a continuous variable. Dispersion estimation and differential methylation assessment were performed as described for the PCa analysis.

In outer fold analyses, we considered a genomic window a DMR when its *p*‐value was ≤ 0.01. For inner fold analyses, a *p*‐value threshold of *p* ≤ 0.05 was used. All analyses were performed with the R programming language and environment v4.4.3 (R Core Team 2023).

A DMR refers to any 300 bp genomic windows with a *p*‐value on the tactic covariate under a specific threshold. A DMR identified in an outer CV‐fold (*f*) by any of the three methods (*m*) is a potential DMR (pDMRs[*f*/*m*], *p* refers to potential, *f* to fold‐specific, *m* refers to method‐specific). These pDMRs[*f*/*m*] were then subjected to an inner CV coherence filter. It was retained as a ‘coherent fold‐specific and method‐specific DMR (cDMRs[*f*/*m*], *c* refers to coherent)’, if it was also found to be significant and showed a consistent direction of methylation change (e.g., always hypermethylated in territorial males compared with floaters, or vice‐versa) across all nine inner CV sub‐folds derived from that specific outer fold's training set. Finally, the cDMRs[*f*/*m*] that were identified in all 10 outer CV‐folds with a consistent direction of methylation change across all 10 folds were retained as cDMRs[*m*].

We assessed the effect of age using the two age classes present in our floater samples, leading to a comparison between five younger (age class 6: > 2 to ≤ 3.5 years) and five older floaters (age class 7: > 3.5 to ≤ 7 years; Table S1). We used the parametric categorical (PCa) test with age class as a covariate (read_count ~ age_class) following the same logic as the main analysis with the difference that these samples were not paired (from the same individuals ‘ID’). Therefore, we left out one sample rather than one ID and performed a two‐step leave‐one‐sample‐out analysis. The Wilcoxon test returned no DMRs passing the threshold, so the age analysis is only based on the PCa method.

### 2.6 Gene Ontology Term Assignment

We assigned differentially methylated genes (DMGs, a gene with an overlapping cDMR[*m*]) to gene ontology (GO) biological process terms using g:Profiler (Raudvere et al. 2019), with *Felis catus* as the reference organism (Ensembl release 114) and the set of genes covered by MBD‐Seq as background, so that terms were assessed against those genes captured in our data (Geeleher et al. 2013), which consisted of 24,380 genes out of the 33,469 annotated genes in the genome (72.8%). Given our low sample size, we did not statistically test our sets of genes for pathway enrichment but assigned them to their associated GO terms and ranked them by an evidence score. We computed this evidence score as the –log_10_ of the Fisher‐combined FDR‐corrected *p*‐value across the three methods; higher scores indicate stronger and more consistent support and used it only to rank terms, not as a statistical test. To avoid any resemblance to a significance threshold, we deliberately report a fixed number of terms (the 80 highest‐ranked, TOP80). Ranked terms were clustered hierarchically using the Jaccard index computed on the DMGs shared across the three methods and assigned to each term.

### 2.7 Biomarker Selection and Predictive Power Estimation

We extracted potential epigenetic biomarkers from the cDMRs[*f*/*m*]. Within each of the 10 outer CV‐folds, ElasticNet regularization (*α* = 0.5, *λ* selected via an internal threefold CV with balanced classes; glmnet package; Tay et al. 2023) was fitted to the TMM‐normalized counts corresponding to its set of ‘cDMRs[*f*/*m*]’. This step selected the most informative DMRs for predicting floater or territorial male phenotype for that specific fold, yielding 10 sets of fold‐specific, method‐specific biomarkers for each analytical stream. To control for the inherent variation in the *λ* parameters estimation, we ran 10 replicates of the glmnet model and selected fold‐specific, method‐specific biomarkers as cDMRs[*f*/*m*] with a non‐zero beta coefficient in all replicates.

We then trained a random forest model (Breiman 2001), using the ranger package (Wright and Ziegler 2017), with default parameters and 1000 trees on the TMM‐normalized counts of these fold‐specific biomarkers, and on the nine individuals in the outer CV‐fold's training set. The predictive power of this model (for tactic classification) was assessed on the left‐out individual pair (the ‘unseen’ data) from the corresponding outer CV‐fold, and measured using average accuracy, area under the curve (AUC), sensitivity and specificity across all 10 outer CV‐folds. The 95% confidence interval (CI) was computed on 500 bootstraps of the random forest model prediction. We defined high‐confidence biomarkers for each analytical method as those DMRs consistently selected by ElasticNet across all 10 outer CV‐folds within one method.

## 3 Results

### 3.1 Body Mass Differences Between Floaters That Remained Floaters and Floaters That Became Territorial Males

The 10 focal males that changed spatial tactic from floater to territorial male showed a pronounced increase in body mass between the two captures, from 46.81 kg (SD = 5.61) as floaters to 54.35 kg (SD = 5.18) as territorial males. This corresponds to a mean within‐individual gain of 7.54 kg (SD = 3.49; paired *t*‐test: *p* < 0.001; Figure S5A). In contrast, eight additional males that remained floaters showed only a minor change in body mass, from 46.66 kg (SD = 4.53) at first capture to 47.59 kg (SD = 3.87) at recapture, corresponding to a mean gain of 0.93 kg (SD = 1.39; paired *t*‐test: *p* = 0.102). On average, the body mass gain of males that became territorial exceeded that of males remaining floaters by 6.62 kg (Welch *t*‐test: *t* = 5.48, *p* < 0.001).

Our methylation analysis is limited to the 10 focal males sampled first as floaters and later as territorial males. Because repeated methylation data were not available from males that remained floaters, we used the two age classes present among the floater samples as an approximate proxy for age‐related differences among floaters (referred to below as the age analysis). Among these samples, younger floaters, that is, age class 6 (> 2.0 to ≤ 3.5 years), weighed 44.70 kg on average (SD = 3.40, *n* = 5), whereas older floaters, that is, age class 7 (> 3.5 to ≤ 7.0 years), weighed 48.92 kg on average (SD = 6.94, *n* = 5), which was not statistically different (Welch *t*‐test: *p* = 0.269; Figure S5B).

### 3.2 Sequencing Results

MBD‐Seq yielded high‐quality data with a mean of 29,001,044 paired‐end clean reads for floaters and 29,541,538 for territorial males (Table S2). Average mapping rates of sequencing reads to the cheetah reference genome were high, 94.3% for floaters (range: 89.3%–98.1%) and 90.8% for territorial males (range: 77.0%–97.9%). All libraries, except one of territorial male T6 with a mapping rate of 77.0%, had mapping rates higher than 88%. The mapping rate difference is without any obvious reason, because read counts and lymphocytes counts were similar to the other samples (Tables S1 and S2).

### 3.3 Methylation Analyses

**FIGURE 2:**
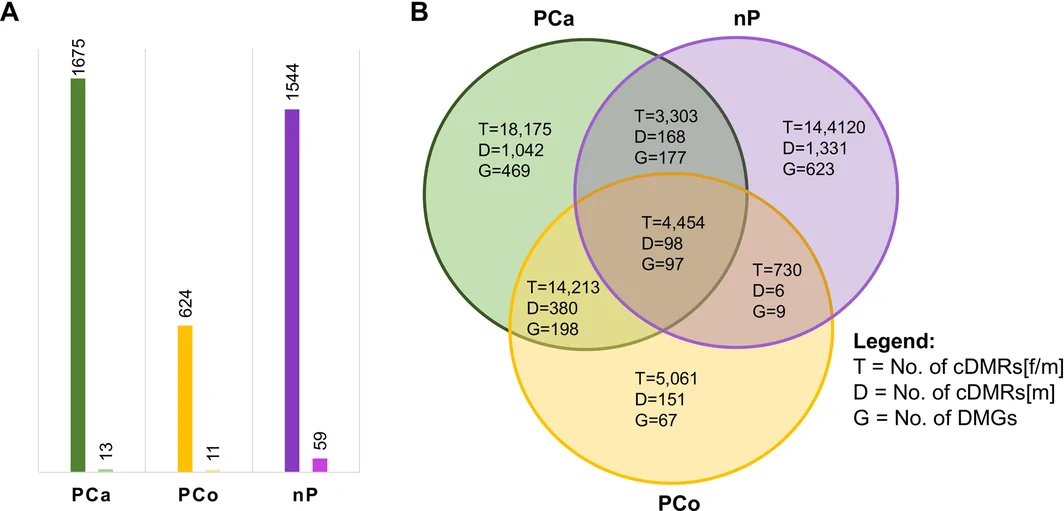
Number of DMRs across methods. (A) The histogram shows the number of DMRs according to their hypermethylation (dark left bars) and hypomethylation (light right bars) in territorial males for the three methods: parametric categorial (PCa, green), parametric continuous (PCo, yellow) and non‐parametric (nP, purple). Most DMRs were hypermethylated in territorial males compared to floaters. (B) The Venn diagram illustrates the numbers and overlaps of DMRs and DMGs identified by the PCa, PCo and nP methods. The values in the diagram correspond to the fold‐specific cDMRs[*f*/*m*] (T), those that are coherent across all folds cDMRs[*m*] (*D*) and the number of DMG (overlapping with cDMRs[*m*]) (*G*) for each method (see Methodical framework in Figure 1, and detailed description in Supporting Information).

To describe the general methylation pattern in our paired experimental design with a low sample size, we focused on methylation patterns coherent across methods and CV‐folds (detailed in Figure 1, Appendix S1: Section I and Figures S1–S3). Most cDMRs[*m*] were hypermethylated in territorial males compared with floaters (Figure 2A, Figure S1). In all three methods, the number of hypermethylated cDMRs[*m*] was higher than expected by chance (PCa: *n* = 1675; PCo: *n* = 624; nP: *n* = 1544; bootstrapped 95% CI: 53%–58% hypermethylated DMRs in randomly selected windows of the same size as the cDMRs[*m*]). We identified 98 cDMRs[*m*] shared across all three methods, while 554 cDMRs[*m*] were detected by at least two methods and were thus considered method‐independent. These method‐independent cDMRs[*m*] overlapped 481 differentially methylated genes (DMGs), of which 97 were detected across all three methods (Figure 2B).

Because every territorial male was previously a floater, age‐related methylation differences could confound the methylation patterns attributed to the spatial tactic‐shift. We therefore compared the two age classes present among the floater samples, that is, younger floaters in age class 6 (> 2.0 to ≤ 3.5 years) and older floaters in age class 7 (> 3.5 to ≤ 7.0 years), as an approximate proxy for age‐related differences among floaters. This age analysis identified 745 age‐associated DMRs, none of which overlapped with the cDMRs[*m*] detected in the spatial tactic‐shift analysis (Table 1). Permutation tests using 1000 random sets of the same size showed that the observed DMR overlap was lower than expected by chance. At the gene level, age‐associated DMRs overlapped 464 DMGs. A minority of these genes also overlapped with tactic‐shift‐associated DMGs: 86 (18.5%) genes with PCa‐associated DMGs, 33 (7.1%) genes with PCo‐associated DMGs, and 74 (15.9%) genes with nP‐associated DMGs (Table 1). Thus, the age and spatial tactic‐shift analyses did not share DMRs, but showed limited overlap after mapping them to genes. In contrast to the DMR‐level result, these gene‐level overlaps were higher than expected under the permutation null. Thus, the age and spatial tactic‐shift analyses did not share DMRs, but showed non‐random convergence after mapping DMRs to genes.

**TABLE 1:** Tactic and age‐associated cDMRs and DMGs comparisons.

Level of comparison	Sensitivity set	Tactic methods	No. age cDMRs or DMGs	No. tactic cDMRs or DMGs	No. overlap between age and tactic cDMRs or DMGs	Permutation mean	Permutation 95% CI	Permutation *p‐value*
DMRs	age_PCa	PCa	745	1688	0	0.93	[0, 3]	1
	age_PCa	PCo	745	635	0	0.32	[0, 2]	1
	age_PCa	nP	745	1603	0	0.87	[0, 3]	1
DMGs	age_PCa	PCa	464	941	86	18.00	[10, 27]	< 0.001
	age_PCa	PCo	464	371	33	9.97	[2, 13]	< 0.001
	age_PCa	nP	464	906	74	17.50	[10, 26]	< 0.001

*Note:* Overlap between age‐associated and spatial tactic‐shift‐associated cDMRs and DMGs. The upper block reports overlaps at the DMR level, while the lower block reports overlaps after assigning cDMRs to genes, that is, as differentially methylated genes (DMGs). The age‐associated set was obtained from the age analysis comparing floaters males in age class 6 (> 2.0 to ≤ 3.5 years) and age class 7 (> 3.5 to ≤ 7.0 years). The spatial tactic‐shift sets correspond to the cDMRs[*m*] or DMGs detected by the three analytical methods: parametric categorical analysis (PCa), parametric continuous analysis (PCo) and non‐parametric analysis (nP). ‘No. age cDMRs or DMGs’ gives the number of age‐associated cDMRs or DMGs, depending on the level of comparison. ‘No. tactic cDMRs or DMGs’ gives the number of spatial tactic‐shift‐associated cDMRs[m] or DMGs in the corresponding method. ‘No. overlap between age and tactic cDMRs or DMGs’ gives the number of cDMRs or DMGs shared between the age and spatial tactic‐shift sets. ‘Permutation mean’ and ‘Permutation 95% CI’ give the expected overlap under 1000 permutations using random sets of the same size. ‘Permutation *p*‐value’ gives the empirical permutation *p*‐value for an overlap equal to or greater than the observed overlap.

### 3.4 Biological Processes

**FIGURE 3:**
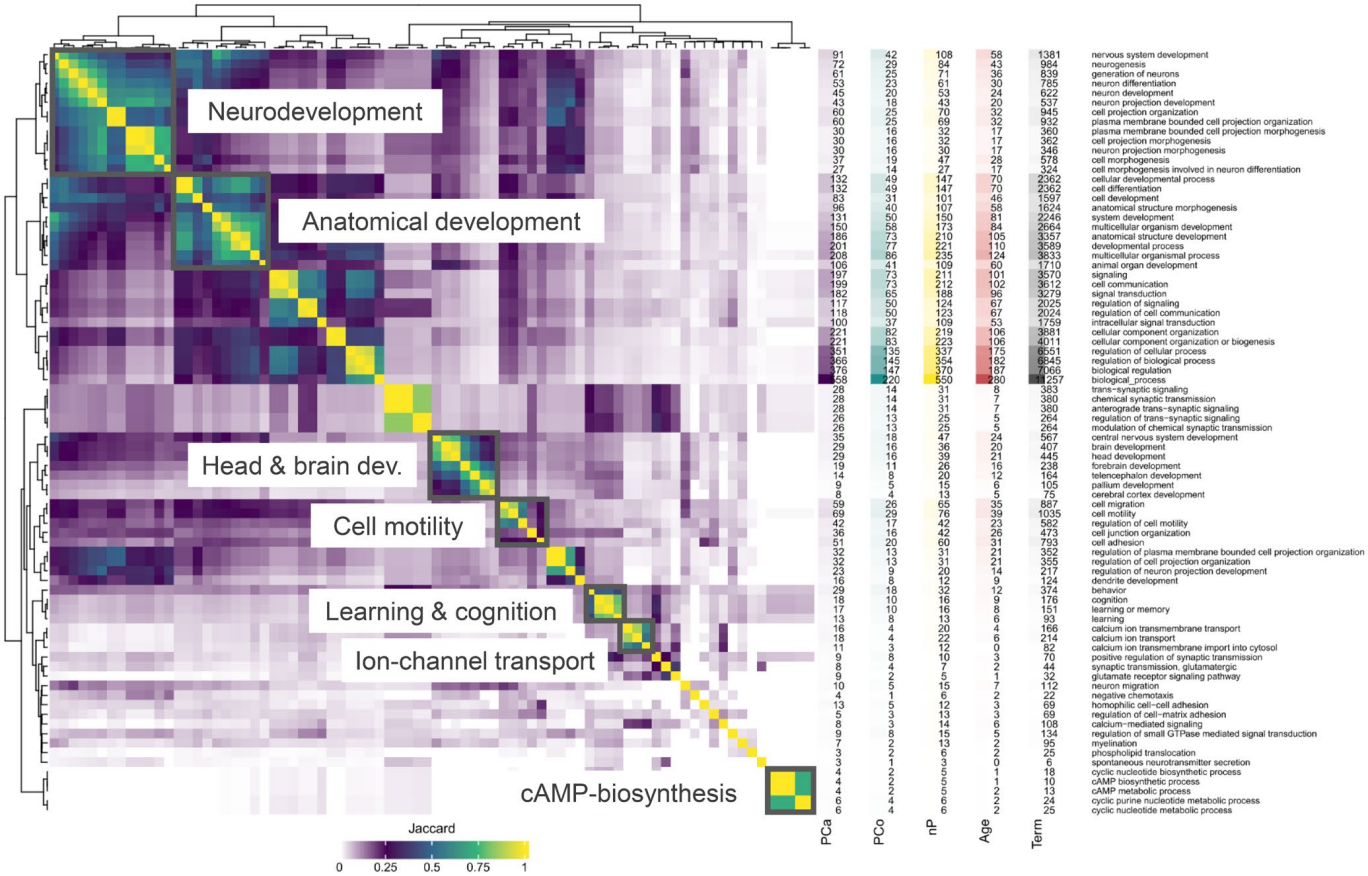
Functional analysis of genes associated with coherent DMRs. Heatmap of the TOP80 GO biological process terms associated with tactic‐shift DMGs and ranked by the tactic‐shift evidence score. Framed clusters indicate seven broad biological process groups identified from shared DMGs across methods. The right‐side annotation panel shows, for each GO term, the number of DMGs detected by the parametric categorical (PCa), parametric continuous (PCo) and non‐parametric (nP) methods, the number of age‐associated DMGs (Age), and the total number of genes assigned to the GO term (Term).

We assigned DMGs to GO biological process terms and ranked the terms using the tactic‐shift evidence score. The 80 highest‐ranked GO terms (TOP80) associated with DMGs detected across the three tactic‐shift methods clustered into seven main biological process groups: neurodevelopment, anatomical development, head and brain development, cell motility, learning and cognition, ion‐channel transport and cAMP biosynthesis (Figure 3). These processes were consistent with the observed phenotypic differences between floaters and territorial males, including morphological changes, reflected by anatomical development and head and brain development, and behavioural changes, reflected by neurodevelopment, learning and cognition, and ion‐channel transport.

The tactic‐shift‐associated TOP80 biological processes showed a more specific and coherent functional signature than the age‐associated TOP80 terms (Figures S3 and S4). While the tactic‐shift‐associated terms were concentrated around developmental, neurodevelopmental, behavioural and signalling‐related processes, the age‐associated TOP80 terms were more broadly distributed across general biological processes, including lymphatic development and regulation, sterol and cholesterol catabolic processes, and cell projection. Some GO terms were shared between the tactic‐shift and age analyses, including anatomical structure morphogenesis and brain development (Figures S3 and S4). The tactic‐associated TOP 80 and full GO term tables are available on Dryad (DOI: 10.5061/dryad.8gtht774k).

### 3.5 Promoter‐Overlapping cDMRs Associated With the Spatial Tactic‐Shift

**FIGURE 4:**
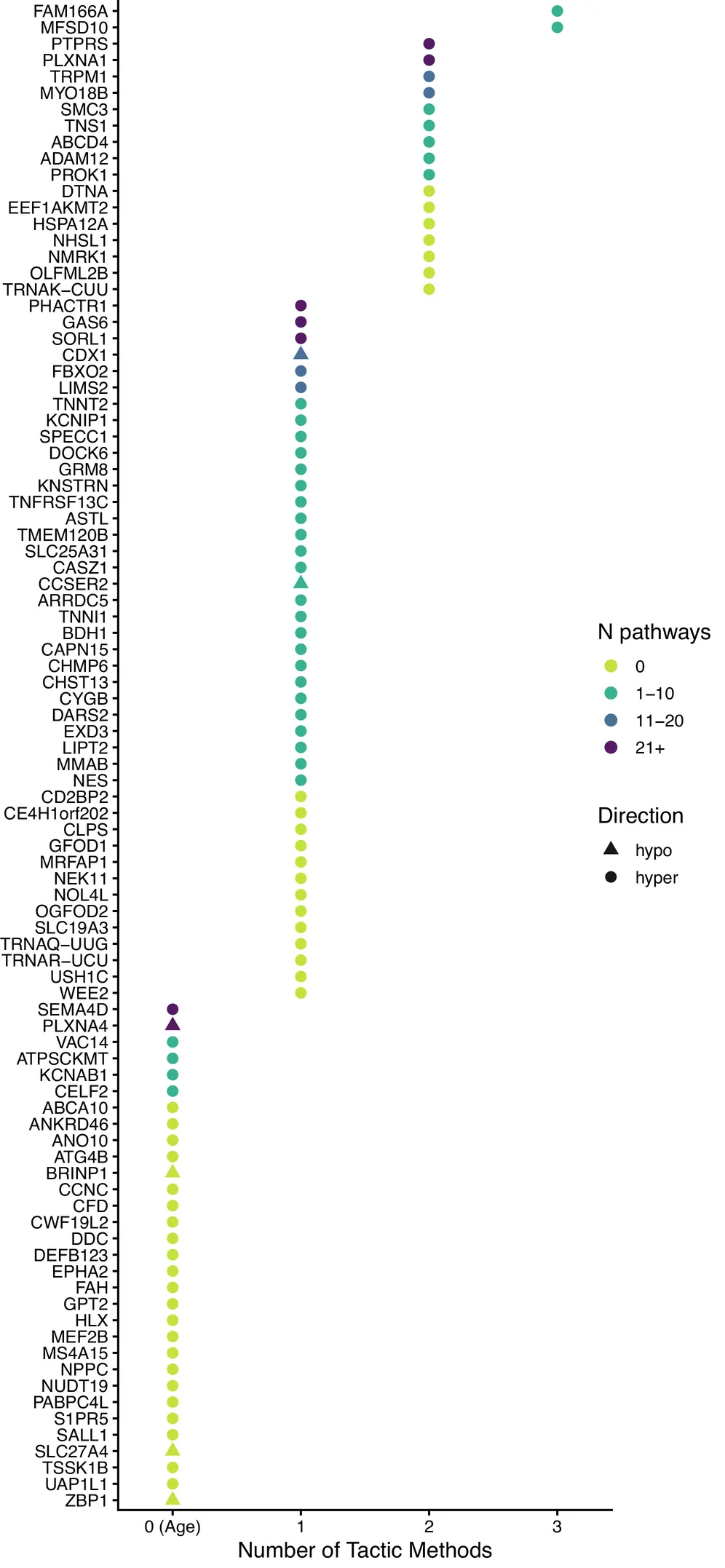
Promoter‐overlapping cDMRs associated with the spatial tactic‐shift and age analysis. Promoter‐overlapping cDMRs[*m*] were detected for 61 tactic‐shift‐associated genes and 31 age‐associated genes, excluding uncharacterized LOC genes. The *y*‐axis lists genes with promoter‐overlapping cDMRs. The *x*‐axis indicates the number of tactic‐shift methods in which promoter‐overlapping cDMRs[*m*] were detected. Genes shown at 0 were detected only in the age analysis, because no tactic‐shift‐associated promoter‐overlapping genes overlapped with the age‐associated promoter set. With the exception of six hypomethylated tactic‐shift‐associated genes, promoter‐overlapping cDMRs[m] were predominantly hypermethylated in territorial males. Point shape indicates methylation direction, and colour indicates the number of TOP80 GO biological process terms assigned to each gene.

Because promoter‐methylation plays a critical role in gene regulation, we focused on cDMRs[*m*] overlapping promoter regions. We detected promoter‐overlapping cDMRs[*m*] in 61 tactic‐shift‐associated genes (104 including uncharacterized LOC genes). The majority of these genes were hypermethylated in territorial males (*n* = 55), whereas only six were hypomethylated (Figure 4). None of the tactic‐shift‐associated genes with promoter‐overlapping cDMRs[*m*] overlapped with the age‐associated promoter set, which consisted of 31 genes (38 genes including LOC genes).

Eighteen method‐independent genes with promoter‐overlapping cDMRs[m] were involved in developmental processes, muscle contraction, cell signalling and cellular metabolism. *MFSD10* and *FAM166A* were detected across all three methods, indicating a particularly consistent promoter‐methylation signal. *LXNA1* and *PTPRS* are involved in neuronal guidance, mediating axon growth cone migration and neurite outgrowth, respectively. *MYO18B*, *DTNA* and *ADAM12* contribute to muscle structure and contraction through sarcomere assembly, membrane stabilization and myoblast fusion. *TNS1*, together with the adhesion‐related functions of *PTPRS* and *DTNA*, mediates cell adhesion and cytoskeletal organization. These processes are complemented by *NHSL1*, which regulates lamellipodial actin dynamics and cell migration as part of the *WAVE* Shell Complex, and *OLFML2B*, a secreted extracellular matrix protein that binds chondroitin sulphate and heparin. *TRPM1* and *PROK1* function in signal transduction and angiogenic or endocrine signalling, respectively, while *HSPA12A*, an atypical member of the *HSP70* family, contributes to angiogenic signalling and endothelial function. *MFSD10* and *ABCD4* are involved in transport and metabolic processes, alongside *NMRK1*, which mediates *NAD*+ biosynthesis via the nicotinamide riboside salvage pathway, and *EEF1AKMT2*, a methyltransferase that modifies translation elongation factor *eEF1A* and thereby fine‐tunes protein synthesis. *SMC3* maintains chromosome cohesion as a core cohesin subunit. *TRNAK*‐*CUU*, a transfer RNA gene, was also detected among the differentially methylated loci, although its regulatory relevance beyond core translational function is unclear. *FAM166A* is an uncharacterized pseudogene.

### 3.6 Coherent DMRs as Biomarkers Discriminating Floater and Territorial Males

We used the left‐out samples from the cross‐validation framework to estimate the predictive power of coherent DMRs for discriminating floaters from territorial males. DMRs derived from the nP method showed the highest predictive performance, classifying the spatial tactic correctly in 78.2% of cases on average (95% bootstrapped CI = 75%–85%; Table S3). The nP‐derived biomarker set also showed the greatest stability across folds, with 19 DMRs consistently selected by ElasticNet across all 10 CV‐folds (Table S4). The PCa and PCo sets reached similar but slightly lower predictive performance, with accuracies of 76.2% for PCa (95% bootstrapped CI = 70%–85%) and 74.0% for PCo (95% bootstrapped CI = 65%–80%). These methods also yielded less stable biomarker sets across folds, with 10 DMRs consistently selected for PCa and three for PCo (Tables S3 and S4).

We compiled a final list of 21 candidate biomarkers that were consistently selected across all CV‐folds by at least one analytical method (Table S4). Of these, seven DMRs overlapped annotated genes: *GLI2*, *ZFYVE28*, *PARVB*, *MUSK*, *ZNF423*, *SHISA6* and *EFCC1*; two DMRs overlapped uncharacterized LOC genes; and 12 DMRs were located in intergenic regions. Because the biomarker analysis was designed to discriminate spatial tactic, age‐associated DMRs were not used for biomarker selection.

## 4 Discussion

Our study explores the association between DNA methylation and phenotypic plasticity in Namibian male cheetahs, a species with low genetic diversity in which regulatory flexibility may play an important role in generating phenotypic variation. We show that the transition from floater to territorial male spatial tactic was accompanied by widespread changes in DNA methylation patterns in PBMCs, with a predominance of hypermethylation in territorial males. Because the same individuals were sampled before and after the transition, these methylation differences cannot be explained by fixed genetic differences among individuals. Instead, they indicate that the observed life‐history transition is associated with changes in gene regulation at the epigenetic level.

The genes overlapping tactic‐shift‐associated DMRs were assigned to biological processes consistent with the morphological, behavioural and spatial changes observed during the transition to territoriality. GO terms related to anatomical development, head development and brain development are consistent with the observed increase in body mass and head size in territorial males. GO terms related to neurodevelopment, learning and cognition, and ion‐channel transport may reflect behavioural changes associated with territoriality, including increased aggression, boldness and altered spatial behaviour. In addition, biological processes related to cAMP biosynthesis and cAMP metabolism point to signalling and energy‐conversion pathways that may be relevant to the physiological and movement changes accompanying the transition from wide‐ranging floaters to territorial males with smaller home ranges. Although the age analysis identified some GO terms shared with the tactic‐shift analysis, including broad developmental processes, the age‐associated signature was less functionally coherent and did not overlap with the tactic‐shift‐associated DMRs. Promoter hypermethylation of several genes may be particularly relevant to the somatic and neuromuscular changes associated with the shift to territoriality. MYO18B regulates muscle‐specific gene expression and actin organization (Latham et al. 2020), SMC3 is involved in chromosomal cohesion and DNA repair (Feeney et al. 2010), and DTNA contributes to muscle integrity and synapse formation (Blake et al. 1998). Similarly, PLXNA1, a gene involved in neuronal guidance, immune regulation and skeletal development (Yoshida et al. 2006), may be linked to both morphological and behavioural changes observed after the transition.

Promoter hypermethylation of PTPRS, a receptor‐type tyrosine phosphatase involved in brain development and the negative regulation of neurotrophic signalling pathways, including PI3K/Akt and MAPK, may also be relevant to neural plasticity and behavioural change during the transition to territoriality. Biological processes related to energy conversion and neurodevelopment are consistent with the ecological contrast between spatial tactics: floaters maintain larger home ranges, whereas territorial males reduce ranging behaviour and display increased aggression. Genes such as TRPM1 and MFSD10, involved in ion‐channel function and energy metabolism, may therefore reflect physiological changes associated with the spatial tactic‐shift. ABCD4, an ATP‐binding transporter involved in cobalamin metabolism, may also be relevant, as homologous pathways have been associated with hyperactivity and aggression in humans (Asheuer et al. 2005).

### 4.1 Interpreting Patterns in Small Sample Size

Because territorial males were older than when they were sampled as floaters, age‐associated methylation differences represent a potential confounder of the spatial tactic‐shift signal. While an overlap existed, the age methylation pattern did not reproduce the methylation pattern observed for the tactic‐shift. The main difference is to be found in the structure of the signal. In the tactic‐shift analysis, the highest‐ranked GO terms formed a coherent functional pattern, clustering around developmental, neurodevelopmental, signalling, ion‐transport, energy‐conversion and cognition‐related processes. These processes are consistent with the observed morphological, behavioural and spatial movement changes of territorial males. In contrast, the age‐associated GO terms were more diffuse and less specifically aligned with the observed phenotypic transition. This suggests that visible physiological or behavioural transitions may depend not only on the presence of methylation differences at single loci, but on their convergence into a structured and functionally coherent regulatory pattern.

Methodologically, this distinction is important for small sample size epigenomic studies. In such systems, convergence of the aggregated signal may be more informative than any single statistic: per‐window estimates are unstable, and a single genome‐wide FDR threshold would exclude nearly all signals. Stability across folds (data sensitivity) and methods (method sensitivity) offers an alternative reliability criterion, one that makes rare but valuable material from a non‐model species usable as evidence while keeping the associated uncertainty explicit rather than hidden inside a single pipeline. In this view, a real effect is separated from background variation by whether many weak signals organize into a consistent functional trend rather than by the value of any single one, in line with the cross‐validation logic used in this study.

A feature of the cross‐validation approach is that it allows prediction to be assessed on unseen data. Because each individual was classified only by a model trained without it, the cross‐validation returns an out‐of‐sample estimate of how well the procedure predicts unseen individuals. This assesses the performance of the fold‐internal selection‐and‐classification procedure, not of any single fixed marker set.

The set of biomarkers reported here was assembled after inspecting all folds and is therefore not itself validated on unseen data. These biomarkers should therefore be treated as candidates for testing in an independent, larger cohort, potentially using cost‐effective assays of these DMRs, such as bisulphite‐PCR. If validated, such markers could help monitor uncollared or elusive individuals, potentially even from non‐invasively collected DNA if sample quality allows (Weyrich et al. 2022). This approach could offer new avenues for assessing population structure, such as floater‐to‐territorial male ratio and habitat saturation, which are directly relevant for conservation management (Durant et al. 2015, 2017; Edwards et al. 2018).

### 4.2 Interpreting PBMC Methylation Signatures

The methylation patterns identified in this study were measured in PBMCs and should therefore be interpreted in the context of blood as the sampled tissue. DNA methylation is highly cell‐type specific and contributes to cell identity and tissue‐specific gene regulation (Smith et al. 2025). Consequently, the cDMRs detected here do not imply that the same methylation changes occur in other tissues involved in the territorial phenotype, such as brain, muscle, or cranial tissues. Tissue‐specific methylation differences have been shown in other species, for example between liver and testes (Weyrich et al. 2020), and similar tissue specificity is likely in cheetahs.

This is particularly important for GO terms related to head development, neurodevelopment or muscle function. Their detection in PBMC‐derived methylation data does not mean that these developmental processes are directly active in blood cells. Rather, PBMC methylation may reflect systemic physiological states associated with the spatial tactic‐shift, because circulating immune cells are exposed to hormones, metabolites, cytokines and other signalling molecules that change with physiology and behaviour. The detected methylation differences may therefore represent a systemic signature of the phenotypic transition, or a record of physiological changes occurring across the organism, rather than direct functional activity of these genes in blood cells.

We also considered whether the methylation differences could reflect major differences in blood cell composition or infection state. Lymphocyte percentages were low and similar between floaters and territorial males, indicating no evidence of acute infection in the investigated cheetahs. In addition, the GO‐term results did not show a dominant immunological signature. Together, these observations make an acute infection or major lymphocyte‐driven cell‐composition effect unlikely, although finer differences in PBMC subtypes cannot be excluded. Finally, MBD‐Seq measures methylation patterns but does not directly measure gene expression. While promoter hypermethylation is often associated with transcriptional repression, the functional consequences of gene‐body methylation are more complex (Jjingo et al. 2012). The DMGs and biological processes reported here should therefore be interpreted as methylation signatures associated with the spatial tactic‐shift, not as direct evidence of altered expression or functional activity in the corresponding tissues.

### 4.3 Cause and Consequence

The identified DNA methylation differences between floaters and territorial males were coherent and reliably distinguished the two spatial tactics. However, they should not be interpreted as evidence that DNA methylation drives the transition from floater to territorial male. Our paired design controls for fixed genetic differences among individuals, because the same males were sampled before and after the spatial tactic shift. However, repeated methylation data were not available from males that remained floaters, and our dataset does not include intermediate samples collected during the transition itself. We therefore cannot determine whether the methylation differences preceded the transition, accompanied it or arose as a consequence of the phenotypic change.

The observed methylation differences are compatible with several causal scenarios. Epigenetic changes could precede and contribute to transcriptional reprogramming associated with territoriality. Equally, the acquisition of a territory may trigger hormonal, metabolic, behavioural or transcription‐factor‐mediated changes that then lead to remodelling of DNA methylation patterns. As cells begin to express new gene programmes and repress others, changes in DNA methylation, histone modification and chromatin structure can follow as part of the normal machinery of gene regulation (Smith et al. 2025). Our data cannot distinguish between these alternatives. We therefore interpret the epigenetic marks identified here as diagnostic of the floater and territorial male spatial tactics, rather than necessarily causative of the transition between them.

More broadly, our study shows that a major within‐individual phenotypic transition in a wild mammal is associated with a coordinated change in DNA methylation, without requiring differences in the underlying DNA sequence among individuals. Such paired sampling of wild individuals before and after a marked change in morphology, behaviour and spatial movement is rare. In a species with low genetic diversity such as the cheetah, this association between phenotypic flexibility and gene‐regulatory variation may be particularly relevant, but future work integrating additional sampling across the transition with transcriptomic, hormonal or functional assays will be necessary to establish temporal ordering and move from association towards mechanism.

## 5 Conclusion

Our study demonstrates a coherent association between DNA methylation and a life‐history transition followed by phenotypic changes in free‐ranging male cheetahs. The identified DMRs, overlapped genes with function in biological processes related to anatomical development, neurodevelopment and energy conversion, mirror the morphological and behavioural differences between floater and territorial males. By leveraging a paired‐sample design in a wild mammal, our results show that a within‐individual transition from one phenotype to another is associated with distinct DNA methylation patterns, without requiring fixed genetic differences among individuals. This regulatory flexibility may contribute to the range of phenotypic responses expressed at the population level, which could be particularly relevant in species with limited genetic diversity.

Future research should explore whether similar mechanisms operate in other threatened species, and whether common epigenetic signatures can serve as biomarkers of adaptive capacity across taxa. The identification of predictive epigenetic markers also enables practical applications in conservation biology, such as non‐invasive monitoring of spatial tactic distributions or population status. Ultimately, integrating epigenetic perspectives into conservation research can refine predictions of extinction risk and guide management strategies in an era of accelerating biodiversity loss.

## Author Contributions

A.W. and B.W. conceptualized the study and acquired the funds. C.V. conceptualized the analyses and curated the data. C.V. and A.W. analysed the data and visualized the results. S.H., J.M. and B.W. conducted the field and lab work in Namibia. D.G. conducted the lab work in Germany and wrote the initial draft. C.V., A.W., B.W. and J.F. wrote the manuscript. All authors read and approved the final manuscript.

## Funding

This study was supported by funds from the Messerli Foundation (Switzerland), Leibniz‐IZW (Germany), the Leibniz Competition Fund (SAW‐2018‐IZW‐3‐EpiRank) and German Centre for Integrative Biodiversity Research (iDiv) Halle‐Jena‐Leipzig (grant sDiv_sInSpEc).

## Disclosure

Benefit‐Sharing Statement: Benefits Generated—Benefits from this research accrue from the sharing of our data and results on public databases as described above.

## Ethics Statement

All procedures were approved by the Leibniz‐IZW Internal Ethics Committee (permit 2002‐04‐01) and authorized by the Ministry of Environment, Forestry and Tourism of Namibia (permit numbers 1300/2008, 1514/2011, 1689/2012, 1813/2013, 1914/2014, 2067/2015, 2208/2017 and RCIV00082018). Samples were transported under CITES permits (Table S1) to the Leibniz‐IZW.

## Conflicts of Interest

The authors declare no conflicts of interest.

## Supporting information


**Figure S1:** Distribution of effect sizes for coherent DMRs from the cross‐validation framework. The plot shows the effect size distribution for all fold‐specific coherent DMRs (cDMRs[*f*/*m*]). Each point represents a single cDMR[*f*/*m*], a region identified as differentially methylated and coherent within one of the outer cross‐validation (CV) folds and one method. The points are coloured based on their overall coherence, which reflects how consistently a region was identified across multiple outer CV‐folds and/or analytical methods. The coherence levels, detailed in the legend, range from regions coherent in at least one fold (‘Inner‐CV Only’, yellow dots) to those coherent across all 10 outer folds and all three methods (‘Outer‐CV + All Methods’, purple lines). The horizontal lines below the distribution summarize the effect size range for all DMRs that meet at least full coherence across one method.
**Figure S2:** Data sensitivity, methodological coherence and characteristics of identified DMRs. (A) Overlap of cDMRs[*f*/*m*] between analytical methods. For each reference method (panels nP [purple], PCa [green], PCo [yellow]), the plot shows the proportion of its fold‐specific coherent DMRs (cDMRs[*f*/*m*]) that are shared with the full set of cDMRs[*f*/*m*] from the other two methods. The cDMRs[*f*/*m*] are grouped by the number of outer CV‐folds they were identified in (*x*‐axis). (B) Number of cDMRs[*f*/*m*] as a function of data sensitivity. For each analytical method, the circles (left *y*‐axis) show the number of cDMRs[*f*/*m*] found in an exact number of outer CV‐folds. The dotted lines connecting the circles (right *y*‐axis) show the cumulative number of unique cDMRs[*f*/*m*] identified in at least that many folds.
**Figure S3:** GO biological process terms detected by each method for spatial tactic‐shift including age. TOP80 GO biological process terms for ranked by the tactic‐shift evidence score, calculated as the Fisher‐combined GO term *p*‐value across PCa, PCo and nP. DM genes associated with the floater to territorial tactic‐shift comparison were functionally assigned to GO terms. Large purple points indicate the combined tactic‐shift evidence score, while smaller points show method‐specific GO term scores (−log_10_ adjusted *p*‐values) for PCa, PCo and nP. Yellow triangles indicate the evidence score of the same GO term in the age comparison among only floaters. Although most TOP80 tactic‐shift‐associated biological processes were also recovered in the age comparison, they generally showed lower evidence scores in age. This suggests convergence at the biological process level despite stronger method‐specific variability at the DMR level.
**Figure S4:** GO biological process terms detected by each method for age including spatial tactic. TOP80 GO biological process terms ranked by the age evidence score per process from the age comparison among only floaters. Age‐associated DM genes among floaters were functionally assigned to GO terms, and each process is shown with its age evidence score. Yellow triangles indicate the age evidence score, while points show the corresponding tactic‐shift‐associated evidence scores for PCa, PCo, nP and the Fisher‐combined tactic‐shift evidence score. Only limited overlap was observed between the TOP80 age‐associated biological processes and the TOP80 tactic‐shift‐associated GO term signals. Taken together with Figure S3, the age‐ranked biological processes appeared functionally less specific, consistent with a more general age‐associated methylation signature. This supports the interpretation that the tactic‐shift‐associated signal is functionally more specific and is not simply overlapping age, nor an age effect.
**Figure S5:** Body mass change in males that remained floaters and males that became territorial. (A) Body mass at first capture and recapture for males that remained floaters (F‐F, *n* = 8) and males that changed spatial tactic from floater to territorial male (F‐T, *n* = 10). Lines connect repeated measurements from the same individual. F‐F males did not significantly change from 46.66 kg (SD = 4.53) at first capture to 47.59 kg (SD = 3.87) at recapture, corresponding to a mean gain of only 0.93 kg at recapture (SD = 1.39; paired *t*‐test: *p* = 0.102). In contrast, F‐T males increased significantly from 46.81 kg (SD = 5.61) as floaters to 54.35 kg (SD = 5.18) as territorial males, corresponding to a mean gain of 7.54 kg (SD = 3.49; paired *t*‐test: *p* < 0.001). The mean body mass gain was 6.62 kg larger in males that became territorial than in males that remained floaters (Welch *t*‐test: *t* = 5.48, *p* < 0.001). (B) Floater body mass of the 10 focal F‐T males used for the age analysis, grouped by age class when capture as floaters. Younger floaters, that is, age class 6 (> 2.0 to ≤ 3.5 years), weighed 44.70 kg on average (SD = 3.40, *n* = 5), whereas older floaters, that is, age class 7 (> 3.5 to ≤ 7.0 years), weighed 48.92 kg on average (SD = 6.94, *n* = 5), which was not statistically different (Welch *t*‐test: *p* = 0.269).
**Table S1:** Individual cheetahs as floaters and territorial males (ID1–ID10) and as floaters captured once and recaptured (ID11–ID18), the time periods between the two captures/blood drawings, age classes, body masses, lymphocytes percentages and Cites permit numbers of the sample transport to Germany.
**Table S2:** Individual sequencing results as raw and clean paired‐end reads, mapped reads and mapping rates for the two spatial tactics of the focal 10 cheetah males (10 IDs), each sampled as floater (F) and again as territory holder (T).
**Table S3:** Predictive performance and coherence of biomarker models for classifying male cheetah spatial tactic.
**Table S4:** Putative epigenetic biomarkers for discriminating floaters from territorial male spatial tactic, including Chromosome, Start, End, GeneID/Intergenic and ElasticNet coefficient ranges for each method (PCa, PCo, nP).

## Data Availability

Sequencing data will be made available on NCBI (https://www.ncbi.nlm.nih.gov/sra/PRJNA1499706) and DMGs and GO terms lists are available on Dryad (DOI: 10.5061/dryad.8gtht774k). The code will be open on github for transparency and reproducibility: https://github.com/vullioud/Vullioud_et_al_2026_Molecular_Ecology_scripts.

## References

[mec70527-bib-0001] Abdolmaleky, H. M. , S. Nohesara , J.‐R. Zhou , and S. Thiagalingam . 2025. “Epigenetics in Evolution and Adaptation to Environmental Challenges: Pathways for Disease Prevention and Treatment.” Epigenomics 17, no. 5: 317–333. 10.1080/17501911.2025.2464529. 39948759 PMC11970782

[mec70527-bib-0002] Asheuer, M. , I. Bieche , I. Laurendeau , et al. 2005. “Decreased Expression of ABCD4 and BG1 Genes Early in the Pathogenesis of X‐Linked Adrenoleukodystrophy.” Human Molecular Genetics 14, no. 10: 1293–1303. 10.1093/hmg/ddi140. 15800013

[mec70527-bib-0003] Beck, D. , E. E. Nilsson , M. Ben Maamar , and M. K. Skinner . 2022. “Environmental Induced Transgenerational Inheritance Impacts Systems Epigenetics in Disease Etiology.” Scientific Reports 12, no. 1: 5452. 10.1038/s41598-022-09336-0. 35440735 PMC9018793

[mec70527-bib-0004] Bird, A. 2002. “DNA Methylation Patterns and Epigenetic Memory.” Genes & Development 16, no. 1: 6–21. 10.1101/gad.947102. 11782440

[mec70527-bib-0005] Blake, D. J. , R. Nawrotzki , N. Y. Loh , D. C. Górecki , and K. E. Davies . 1998. “β‐Dystrobrevin, a Member of the Dystrophin‐Related Protein Family.” Proceedings of the National Academy of Sciences of the United States of America 95, no. 1: 241–246. 10.1073/pnas.95.1.241. 9419360 PMC18188

[mec70527-bib-0006] Bosse, M. , and S. van Loon . 2022. “Challenges in Quantifying Genome Erosion for Conservation.” Frontiers in Genetics 13: 960958. 10.3389/fgene.2022.960958. 36226192 PMC9549127

[mec70527-bib-0007] Breiman, L. 2001. “Random Forests.” Machine Learning 45, no. 1: 5–32. 10.1023/A:1010933404324.

[mec70527-bib-0008] Caro, T. M. 1994. Cheetahs of the Serengeti Plains: Group Living in an Asocial Species. University of Chicago Press.

[mec70527-bib-0009] Castro‐Prieto, A. , B. Wachter , and S. Sommer . 2011. “Cheetah Paradigm Revisited: MHC Diversity in the World's Largest Free‐Ranging Population.” Molecular Biology and Evolution 28, no. 4: 1455–1468. 10.1093/molbev/msq330. 21183613 PMC7187558

[mec70527-bib-0010] Danecek, P. , J. K. Bonfield , J. Liddle , et al. 2021. “Twelve Years of SAMtools and BCFtools.” GigaScience 10, no. 2: giab008. 10.1093/gigascience/giab008. 33590861 PMC7931819

[mec70527-bib-0011] Durant, S. M. , M. S. Becker , S. Creel , et al. 2015. “Developing Fencing Policies for Dryland Ecosystems.” Journal of Applied Ecology 52, no. 3: 544–551. 10.1111/1365-2664.12415.

[mec70527-bib-0012] Durant, S. M. , N. Mitchell , R. Groom , et al. 2017. “The Global Decline of Cheetah *Acinonyx jubatus* and What It Means for Conservation.” Proceedings of the National Academy of Sciences of the United States of America 114, no. 3: 528–533. 10.1073/pnas.1611122114. 28028225 PMC5255576

[mec70527-bib-0013] Edwards, S. , M. Fischer , B. Wachter , and J. Melzheimer . 2018. “Coping With Intrasexual Behavioral Differences: Capture–Recapture Abundance Estimation of Male Cheetah.” Ecology and Evolution 8, no. 18: 9171–9180. 10.1002/ece3.4410. 30377492 PMC6194303

[mec70527-bib-0014] Feeney, K. M. , C. W. Wasson , and J. L. Parish . 2010. “Cohesin: A Regulator of Genome Integrity and Gene Expression.” Biochemical Journal 428, no. 2: 147–161. 10.1042/BJ20100151. 20462401

[mec70527-bib-0015] Fortes, G. G. , and J. L. A. Paijmans . 2015. “Analysis of Whole Mitogenomes From Ancient Samples.” In Whole Genome Amplification: Methods and Protocols, edited by T. Kroneis , 179–195. Springer. 10.1007/978-1-4939-2990-0_13. 26374318

[mec70527-bib-0016] Geeleher, P. , L. Hartnett , L. J. Egan , A. Golden , R. A. Raja Ali , and C. Seoighe . 2013. “Gene‐Set Analysis Is Severely Biased When Applied to Genome‐Wide Methylation Data.” Bioinformatics 29, no. 15: 1851–1857. 10.1093/bioinformatics/btt311. 23732277

[mec70527-bib-0017] Guerrero, T. P. , J. Fickel , S. Benhaiem , and A. Weyrich . 2020. “Epigenomics and Gene Regulation in Mammalian Social Systems.” Current Zoology 66, no. 3: 307–319. 10.1093/cz/zoaa005. 32440291 PMC7233906

[mec70527-bib-0018] Heinrich, S. K. , H. Hofer , A. Courtiol , et al. 2017. “Cheetahs Have a Stronger Constitutive Innate Immunity Than Leopards.” Scientific Reports 7, no. 1: 44837. 10.1038/srep44837. 28333126 PMC5363065

[mec70527-bib-0019] Jablonka, E. , and G. Raz . 2009. “Transgenerational Epigenetic Inheritance: Prevalence, Mechanisms, and Implications for the Study of Heredity and Evolution.” Quarterly Review of Biology 84, no. 2: 2. 10.1086/598822. 19606595

[mec70527-bib-0020] Jjingo, D. , A. B. Conley , S. V. Yi , V. V. Lunyak , and I. K. Jordan . 2012. “On the Presence and Role of Human Gene‐Body DNA Methylation.” Oncotarget 3, no. 4: 462–474. 10.18632/oncotarget.497. 22577155 PMC3380580

[mec70527-bib-0021] Langmead, B. , and S. L. Salzberg . 2012. “Fast Gapped‐Read Alignment With Bowtie 2.” Nature Methods 9, no. 4: 357–359. 10.1038/nmeth.1923. 22388286 PMC3322381

[mec70527-bib-0022] Latham, S. L. , N. Weiß , K. Schwanke , et al. 2020. “Myosin‐18B Regulates Higher‐Order Organization of the Cardiac Sarcomere Through Thin Filament Cross‐Linking and Thick Filament Dynamics.” Cell Reports 32, no. 9: 108090. 10.1016/j.celrep.2020.108090. 32877672

[mec70527-bib-0023] Lea, A. J. , J. Altmann , S. C. Alberts , and J. Tung . 2016. “Resource Base Influences Genome‐Wide DNA Methylation Levels in Wild Baboons ( *Papio cynocephalus* ).” Molecular Ecology 25, no. 8: 1681–1696. 10.1111/mec.13436. 26508127 PMC4846536

[mec70527-bib-0024] Martin, B. K. 2007. “Transcriptional Control of Complement Receptor Gene Expression.” Immunologic Research 39, no. 1–3: 146–159. 10.1007/s12026-007-0078-z. 17917062

[mec70527-bib-0025] Martin, M. 2011. “Cutadapt Removes Adapter Sequences From High‐Throughput Sequencing Reads.” EMBnet.Journal 17, no. 1: 1. 10.14806/ej.17.1.200.

[mec70527-bib-0026] Melzheimer, J. , S. K. Heinrich , B. Wasiolka , et al. 2020. “Communication Hubs of an Asocial Cat Are the Source of a Human–Carnivore Conflict and Key to Its Solution.” Proceedings of the National Academy of Sciences of the United States of America 117, no. 52: 33325–33333. 10.1073/pnas.2002487117. 33288693 PMC7776775

[mec70527-bib-0027] Melzheimer, J. , S. Streif , B. Wasiolka , et al. 2018. “Queuing, Takeovers, and Becoming a Fat Cat: Long‐Term Data Reveal Two Distinct Male Spatial Tactics at Different Life‐History Stages in Namibian Cheetahs.” Ecosphere 9, no. 6: e02308. 10.1002/ecs2.2308.

[mec70527-bib-0028] Pečnerová, P. , G. Garcia‐Erill , X. Liu , et al. 2021. “High Genetic Diversity and Low Differentiation Reflect the Ecological Versatility of the African Leopard.” Current Biology 31, no. 9: 1862–1871.e5. 10.1016/j.cub.2021.01.064. 33636121

[mec70527-bib-0029] Putri, G. H. , S. Anders , P. T. Pyl , J. E. Pimanda , and F. Zanini . 2022. “Analysing High‐Throughput Sequencing Data in Python With HTSeq 2.0.” Bioinformatics 38, no. 10: 2943–2945. 10.1093/bioinformatics/btac166. 35561197 PMC9113351

[mec70527-bib-0030] R Core Team . 2023. “R: The R Project for Statistical Computing.” https://www.r‐project.org/.

[mec70527-bib-0031] Raudvere, U. , L. Kolberg , I. Kuzmin , et al. 2019. “G:Profiler: A Web Server for Functional Enrichment Analysis and Conversions of Gene Lists (2019 Update).” Nucleic Acids Research 47, no. W1: W191–W198. 10.1093/nar/gkz369. 31066453 PMC6602461

[mec70527-bib-0051] Robinson, M. D. , D. J. McCarthy , and G. K. Smyth . 2010. “edgeR: A Bioconductor Package for Differential Expression Analysis of Digital Gene Expression Data.” Bioinformatics 26, no. 1: 1. 10.1093/bioinformatics/btp616. 19910308 PMC2796818

[mec70527-bib-0032] Ryu, T. , H. D. Veilleux , P. L. Munday , I. Jung , J. M. Donelson , and T. Ravasi . 2020. “An Epigenetic Signature for Within‐Generational Plasticity of a Reef Fish to Ocean Warming.” Frontiers in Marine Science 7: 284. 10.3389/fmars.2020.00284.

[mec70527-bib-0033] Shaw, R. E. , K. A. Farquharson , M. W. Bruford , et al. 2025. “Global Meta‐Analysis Shows Action Is Needed to Halt Genetic Diversity Loss.” Nature 638, no. 8051: 704–710. 10.1038/s41586-024-08458-x. 39880948 PMC11839457

[mec70527-bib-0034] Skinner, M. K. 2007. “Endocrine Disruptors and Epigenetic Transgenerational Disease Etiology.” Pediatric Research 61, no. 7: 48–50. 10.1203/pdr.0b013e3180457671. 17413841 PMC5940327

[mec70527-bib-0035] Smith, Z. D. , S. Hetzel , and A. Meissner . 2025. “DNA Methylation in Mammalian Development and Disease.” Nature Reviews Genetics 26, no. 1: 7–30. 10.1038/s41576-024-00760-8. 39134824

[mec70527-bib-0036] Stearns, S. C. 1989. “Trade‐Offs in Life‐History Evolution.” Functional Ecology 3, no. 3: 259–268. 10.2307/2389364.

[mec70527-bib-0037] Szyf, M. 2009. “Epigenetics, DNA Methylation, and Chromatin Modifying Drugs.” Annual Review of Pharmacology and Toxicology 49: 243–263. 10.1146/annurev-pharmtox-061008-103102. 18851683

[mec70527-bib-0038] Szyf, M. 2011. “The Early Life Social Environment and DNA Methylation.” Epigenetics 6, no. 8: 971–978. 10.4161/epi.6.8.16793. 21772123 PMC3359487

[mec70527-bib-0039] Szyf, M. , and J. Bick . 2013. “DNA Methylation: A Mechanism for Embedding Early Life Experiences in the Genome.” Child Development 84, no. 1: 49–57. 10.1111/j.1467-8624.2012.01793.x. 22880724 PMC4039199

[mec70527-bib-0040] Tay, J. K. , B. Narasimhan , and T. Hastie . 2023. “Elastic Net Regularization Paths for All Generalized Linear Models.” Journal of Statistical Software 106: 1–31. 10.18637/jss.v106.i01. 37138589 PMC10153598

[mec70527-bib-0041] Thalwitzer, S. , B. Wachter , N. Robert , et al. 2010. “Seroprevalences to Viral Pathogens in Free‐Ranging and Captive Cheetahs ( *Acinonyx jubatus* ) on Namibian Farmland.” Clinical and Vaccine Immunology 17, no. 2: 232–238. 10.1128/CVI.00345-09. 19955325 PMC2815525

[mec70527-bib-0042] Volanakis, J. E. 1995. “Transcriptional Regulation of Complement Genes.” Annual Review of Immunology 13: 277–305. 10.1146/annurev.iy.13.040195.001425. 7612224

[mec70527-bib-0043] Vullioud, C. , S. Benhaiem , D. Meneghini , et al. 2024. “Epigenetic Signatures of Social Status in Wild Female Spotted Hyenas ( *Crocuta crocuta* ).” Communications Biology 7, no. 1: 313. 10.1038/s42003-024-05926-y. 38548860 PMC10978994

[mec70527-bib-0044] West‐Eberhard, M. J. 1989. “Phenotypic Plasticity and the Origins of Diversity.” Annual Review of Ecology, Evolution, and Systematics 20: 249–278. 10.1146/annurev.es.20.110189.001341.

[mec70527-bib-0045] Weyrich, A. , T. P. Guerrero‐Altamirano , S. Yasar , G. Á. Czirják , B. Wachter , and J. Fickel . 2022. “First Steps Towards the Development of Epigenetic Biomarkers in Female Cheetahs ( *Acinonyx jubatus* ).” Life 12, no. 6: 920. 10.3390/life12060920. 35743950 PMC9225391

[mec70527-bib-0046] Weyrich, A. , M. Jeschek , K. T. Schrapers , et al. 2018. “Diet Changes Alter Paternally Inherited Epigenetic Pattern in Male Wild Guinea Pigs.” Environmental Epigenetics 4, no. 2: dvy011. 10.1093/eep/dvy011. 29992049 PMC6031029

[mec70527-bib-0047] Weyrich, A. , D. Lenz , M. Jeschek , et al. 2016. “Paternal Intergenerational Epigenetic Response to Heat Exposure in Male Wild Guinea Pigs.” Molecular Ecology 25, no. 8: 1729–1740. 10.1111/mec.13494. 26686986

[mec70527-bib-0048] Weyrich, A. , S. Yasar , D. Lenz , and J. Fickel . 2020. “Tissue‐Specific Epigenetic Inheritance After Paternal Heat Exposure in Male Wild Guinea Pigs.” Mammalian Genome 31, no. 5–6: 157–169. 10.1007/s00335-020-09832-6. 32285146 PMC7369130

[mec70527-bib-0049] Wright, M. N. , and A. Ziegler . 2017. “Ranger: A Fast Implementation of Random Forests for High Dimensional Data in C++ and R.” Journal of Statistical Software 77: 1–17. 10.18637/jss.v077.i01.

[mec70527-bib-0050] Yoshida, Y. , B. Han , M. Mendelsohn , and T. M. Jessell . 2006. “PlexinA1 Signaling Directs the Segregation of Proprioceptive Sensory Axons in the Developing Spinal Cord.” Neuron 52, no. 5: 775–788. 10.1016/j.neuron.2006.10.032. 17145500 PMC1847564

