# Supplementary material for: Epigenetic Changes Associated With a Phenotypic Transition in Namibian Cheetahs
Source: Mol Ecol. 2026 Sep 7;35(17):e70527. doi: 10.1111/mec.70527 (PMC13548066; doi:10.1111/mec.70527)
Supplement: Supplementary file 1 [file MEC-35-e70527-s001.docx]

**Supplementary Material**

**Content:**

I. **Methodical framework**
II. **Sensitivity analysis incl. Supplementary Figures S1-2**
III. **Supplementary Figures S3-5**
IV. **Supplementary Tables S1-4**
I. **Multi-method cross-validation: Differential methylation analysis within a cross-validation framework using three statistical methods**

Our dataset is characterized by a high dimensionality, with 1,381,830 genomic windows, and a relatively low sample size, with 20 samples from 10 paired individuals. Our main goal was to identify robust and generalizable epigenetic patterns associated with a complex set of behaviours categorized as "spatial tactic". Standard analytical approaches, such as a full-set analysis where each window is analysed independently and then filtered using a threshold on the False Discovery Rate (FDR) correction of p-values, face significant limitations in this context. FDR procedures, while designed to control false positives across multiple tests, can be overly conservative or unstable with high-dimensionality (number of genomic windows) low-sample-size (number of biological replicate) data, potentially masking true biological signals (Putri et al. 2022). To illustrate the limitation of applying a standard FDR correction to our full dataset (for example using a model ~tactic + ID yielded very few significant DMRs (4 windows at FDR ≤ 0.05; 7 at FDR ≤ 0.1, with Benjamini-Hochberg (BH) correction), rendering this approach suboptimal for uncovering potentially widespread, albeit subtle, epigenetic changes. On the other hand, simply lowering the p-value threshold without robust control would inflate the risk of false positives and overfitting, especially given the structure of our data.

To address these challenges and to identify coherent DMRs consistently associated with the shift in phenotype, we implemented a cross-validation (CV) framework (see Figure 1). As our samples were paired, a natural approach was a leave-one-ID-out (LoIDo) CV, allowing for the use of a less stringent initial p-value threshold while controlling for coherence across the CV-folds. This approach is also informative as it simulates the common field scenario where an individual might not be sampled or a sample might not be successfully sequenced.

Furthermore, because the two "phenotypes" are a human-defined category aggregating various behavioural and physiological changes (e.g., spatial movements, body mass, aggressive behaviour), and because different statistical methods have varying sensitivities and assumptions, we analysed the data within each CV-fold using three statistical methods (inspired by the multiverse approach by (Steegen et al. 2016): 1) a non-parametric paired Wilcoxon test (nP), sensitive to consistent rank changes; 2) a parametric negative binomial generalized linear model (GLM) with "phenotype" as a categorical predictor (PCa); and 3) a parametric negative binomial GLM with "body mass" as a continuous proxy for phenotype (PCo). This cross-validation, multi-method approach allows us to:

(1) Evaluate the data-sensitivity of our results: how sensitive are the findings to the inclusion/exclusion of specific individuals (via comparison between folds).
(2) Assess the method-sensitivity of our results: how sensitive are the findings to the statistical approaches used to get them (via comparison between methods).

(3) Enhance confidence in coherent findings: DMRs identified consistently across CV-folds and by different analytical methods are more likely to represent genuine biological signals than statistical artefacts or idiosyncrasies of a particular model or subset of data.

(4) Provide an unbiased estimation of predictive power for potential biomarkers using the left-out ID of each fold as unseen data.

This comprehensive strategy, by explicitly exploring data and method dependencies and prioritizing coherent results, aims to identify robust and generalizable epigenetic signatures associated with the cheetahs' spatial tactic transition, prioritizing more comprehensive insights over a single analysis pathway.

II. **Sensitivity analysis**

**Fold-specific analyses reveal data and method sensitivity**

Combining cross-validation and different modelling approaches (see methods), our framework allows us to assess data and method sensitivity. The ten outer CV-folds revealed 60,356 internally coherent, fold-specific, method-specific DMRs, termed “cDMRs[f/m]” (Figure S1). These are regions that were significant and internally coherent in at least one outer CV-fold (outer: p ≤ 0.01, & inner: p ≤ 0.05 and consistent effect direction across all sub-folds, Figure 1).

***Figure S1: Distribution of effect sizes for coherent DMRs from the cross-validation framework.*** *The plot shows the effect size distribution for all fold-specific coherent DMRs (cDMRs[f/m]). Each point represents a single cDMR[f/m], a region identified as differentially methylated and coherent within one of the outer cross-validation (CV) folds and one method. The points are colored based on their overall coherence, which reflects how consistently a region was identified across multiple outer CV-folds and/or analytical methods. The coherence levels, detailed in the legend, range from regions coherent in at least one fold ("Inner-CV Only", yellow dots) to those coherent across all 10 outer folds and all three methods ("Outer-CV + All Methods", purple lines). The horizontal lines below the distribution summarize the effect size range for all DMRs that meet at least full coherence across one method.*

The choice of statistical method influenced which cDMRs[f/m] were identified.  While the two parametric methods (PCa and PCo) showed considerable concordance (over >98% between the cDMRs[f/m] found in all folds in one method against all cDMRs[f/m] of the other methods), the non-parametric nP method yielded a more distinct set of initial findings with a maximum of 50% of shared DMRs with the other sets (Figure S2A). Interestingly, the maximum overlap between nP and the other two methods is not in 10 nP sets, but in the 9 fold. This suggests that regions exhibiting strong, but not fully rank-based differences were often captured as significant by effect-size sensitive parametric approaches.

**A**

**B**

***Figure S2: Data sensitivity, methodological coherence, and characteristics of identified DMRs. A)*** *Overlap of cDMRs[f/m] between analytical methods. For each reference method (panels nP (purple), PCa (green), PCo (yellow)), the plot shows the proportion of its fold-specific coherent DMRs (cDMRs[f/m]) that are shared with the full set of cDMRs[f/m] from the other two methods. The cDMRs[f/m] are grouped by the number of outer CV-folds they were identified in (x-axis).* ***B)*** *Number of cDMRs[f/m] as a function of data sensitivity. For each analytical method, the circles (left y-axis) show the number of cDMRs[f/m] found in an exact number of outer CV-folds. The dotted lines connecting the circles (right y-axis) show the cumulative number of unique cDMRs[f/m] identified in at least that many folds.*

The resulting cDMRs[f/m] were highly dependent on the specific individuals included in each outer CV-fold and the type of method used. Of the cDMRs[f/m] identified 46% (28,053) were found only with a unique combination of data and method. All methods displayed a strong data-sensitivity. Depending on the method used the total number of cDMRs[f/m] found varied between 22,907 for nP, 40,145 for PCa and 24,458 for PCo, most of them (nP: 19,920 (87%); PCa: 18,417 (46%); PCo: 15,420 (63%)) were found only in one fold, indicating that they were not observed as soon as one ID was permuted (Figure S3B). On the other end, only a small proportion of the DMRs (nP: 1,603 (7.0%); PCa: 1,688 (4.2%); PCo: 635 (2.6%)) were fully coherent (i.e. across all outer CV-folds, further called coherent DMRs per method (m), “cDMRs[m]”, Figure S3B, single circles, at 10 fold).

The partial overlap among cDMRs[f/m] stems from an inherent data-sensitivity combined with the distinct analytical sensitivities of the methods. While the non-parametric nP method identifies regions with highly consistent rank-based changes even with small effect sizes, the parametric PCa and PCo methods are more sensitive to the magnitude of change, preferentially identifying DMRs with larger effect sizes (Figure S2). These two factors—data variability and method-specific sensitivity—explain why increasing the coherence requirement forces the methods to converge on the most stable signals. As stringency increases by requiring a DMR’s presence in more CV-folds, the proportional overlap between the methods grows (Figure S3B). This dynamic demonstrates that a signal coherent within a single data subset may not be globally stable, highlighting the importance of the final filtering step to isolate the most robust epigenetic signatures (cDMRs[m]).

**III. Supplementary Figures**

***Figure S3: GO biological process terms detected by each method for spatial tactic-shift including age.*** *TOP80 GO biological process terms for ranked by the tactic-shift evidence score, calculated as the Fisher-combined GO term p-value across PCa, PCo, and nP. DM genes associated with the floater to territorial tactic-shift comparison were functionally assigned to GO terms. Large purple points indicate the combined tactic-shift evidence score, while smaller points show method-specific GO term scores (-log10 adjusted p-values) for PCa, PCo, and nP. Yellow triangles indicate the evidence score of the same GO term in the age comparison among only floaters. Although most TOP80 tactic-shift-associated biological processes were also recovered in the age comparison, they generally showed lower evidence scores in age. This suggests convergence at the biological process level despite stronger method-specific variability at the DMR level.*

 ***Figure S4: GO biological process terms detected by each method for age including spatial tactic.*** *TOP80 GO biological process terms ranked by the age evidence score per process from the age comparison among only floaters. Age-associated DM genes among floaters were functionally assigned to GO terms, and each process is shown with its age evidence score. Yellow triangles indicate the age evidence score, while points show the corresponding tactic-shift-associated evidence scores for PCa, PCo, nP, and the Fisher-combined tactic-shift evidence score. Only limited overlap was observed between the TOP80 age-associated biological processes and the TOP80 tactic-shift-associated GO term signals. Taken together with Figure S3, the age-ranked biological processes appeared functionally less specific, consistent with a more general age-associated methylation signature. This supports the interpretation that the tactic-shift-associated signal is functionally more specific and is not simply overlapping age, nor an age effect.*

***Figure S5: Body mass change in males that remained floaters and males that became territorial. A)*** *Body mass at first capture and recapture for males that remained floaters (F-F, n = 8) and males that changed spatial tactic from floater to territorial male (F-T, n = 10). Lines connect repeated measurements from the same individual. F-F males did not significantly change from 46.66 kg (SD = 4.53) at first capture to 47.59 kg (SD = 3.87) at recapture, corresponding to a mean gain of only 0.93 kg at recapture (SD = 1.39; paired t-test: p = 0.102). In contrast, F-T males increased significantly from 46.81 kg (SD = 5.61) as floaters to 54.35 kg (SD = 5.18) as territorial males, corresponding to a mean gain of 7.54 kg (SD = 3.49; paired t-test: p < 0.001). The mean body mass gain was 6.62 kg larger in males that became territorial than in males that remained floaters (Welch t-test: t = 5.48, p < 0.001).* ***B)*** *Floater body mass of the ten focal F-T males used for the age analysis, grouped by age class when capture as floaters. Younger floaters, i.e. age class 6 (>2.0–≤3.5 years), weighed 44.70 kg on average (SD = 3.40, n = 5), whereas older floaters, i.e. age class 7 (>3.5–≤7.0 years), weighed 48.92 kg on average (SD = 6.94, n = 5), which was not statistically different (Welch t-test: p = 0.269).*

**IV. Supplementary Tables**

**Table S1:** Individual cheetahs as floaters and territorial males (ID1-ID10) and as floaters captured once and recaptured (ID11-ID18), the time periods between the two captures/blood drawings, age classes, body masses, lymphocytes percentages and Cites permit numbers of the sample transport to Germany.

|  | **ID** | **Spatial tactic** | **Age class*** | **Months between samples** | **Body mass [kg]** | **%Lymphocytes** | **Cites permit numbers** |
| --- | --- | --- | --- | --- | --- | --- | --- |
| 1 | ID1 | Floater | 6 | 11,41 | 40,50 | 12,00 | E-06496/13 |
| 2 | ID1 | Territorial | 7 |  | 43,50 | 18,00 | E-01748/16 |
| 3 | ID2 | Floater | 6 | 15,09 | 47,50 | 26,00 | E-06496/13 |
| 4 | ID2 | Territorial | 7 |  | 59,00 | 10,00 | E-06496/13 |
| 5 | ID3 | Floater | 6 | 11,41 | 47,00 | 20,00 | E-06496/13 |
| 6 | ID3 | Territorial | 7 |  | 52,00 | 20,00 | E-01748/16 |
| 7 | ID4 | Floater | 7 | 38,43 | 55,50 | 20,00 | E-05403/11 |
| 8 | ID4 | Territorial | 8 |  | 61,00 | 12,00 | E-06496/13 |
| 9 | ID5 | Floater | 7 | 25,02 | 54,00 | 14,00 | E-07371/18 |
| 10 | ID5 | Territorial | 7 |  | 58,00 | 19,00 | E-07371/18 |
| 11 | ID6 | Floater | 7 | 39,58 | 38,00 | 7,00 | E-01748/16 |
| 12 | ID6 | Territorial | 8 |  | 50,00 | 8,00 | E-07371/18 |
| 13 | ID7 | Floater | 6 | 59,90 | 41,50 | 8,00 | E-06496/13 |
| 14 | ID7 | Territorial | 8 |  | 52,00 | 12,00 | E-01881/21 |
| 15 | ID8 | Floater | 6 | 6,15 | 47,00 | 1,00 | E-06496/13 |
| 16 | ID8 | Territorial | 7 |  | 55,50 | 11,00 | E-06496/13 |
| 17 | ID9 | Floater | 7 | 18,84 | 50,00 | 7,00 | E-05403/11 |
| 18 | ID9 | Territorial | 7 |  | 54,50 | 4,00 | E-06496/13 |
| 19 | ID10 | Floater | 7 | 31,23 | 47,10 | 18,00 | E-05403/11 |
| 20 | ID10 | Territorial | 7 |  | 58,00 | 11,00 | E-05403/11 |
| 21 | ID11 | Floater First Capture | 6 | 17,79 | 41,00 | 33,00 | E-05403/11 |
| 22 | ID11 | Floater Recapture | 7 |  | 43,00 | 10,00 | E-05403/11 |
| 23 | ID12 | Floater First Capture | 7 | 18,41 | 52,00 | 14,00 | E-06496/13 |
| 24 | ID12 | Floater Recapture | 7 |  | 54,00 | NA | E-01748/16 |
| 25 | ID13 | Floater First Capture | 6 | 12,46 | 41,50 | 8,00 | E-06496/13 |
| 26 | ID13 | Floater Recapture | 7 |  | 44,50 | 8,00 | E-01748/16 |
| 27 | ID14 | Floater First Capture | 7 | 10,22 | 47,00 | 9,00 | E-01748/16 |
| 28 | ID14 | Floater Recapture | 7 |  | 47,00 | 10,00 | E-01748/16 |
| 29 | ID15 | Floater First Capture | 7 | 10,22 | 48,00 | 5,00 | E-01748/16 |
| 30 | ID15 | Floater Recapture | 7 |  | 49,00 | 10,00 | E-01748/16 |
| 31 | ID16 | Floater First Capture | 6 | 12,85 | 42,20 | 10,00 | E-4563/05 |
| 32 | ID16 | Floater Recapture | 7 |  | 43,20 | 15,00 | E-4563/05 |
| 33 | ID17 | Floater First Capture | 7 | 7,59 | 50,60 | 2,00 | E-05403/11 |
| 34 | ID17 | Floater Recapture | 7 |  | 50,00 | NA | E-05403/11 |
| 35 | ID18 | Floater First Capture | 7 | 30,08 | 51,00 | NA | E-01748/16 |
| 36 | ID18 | Floater Recapture | 7 |  | 50,00 | 1,00 | E-07371/18 |

**Age class 6: >2.0 - ≤3.5 years, age class 7: >3.5- ≤7.0 years, age class 8: >7.0 years; NA: data not available.*

**Table S2:** Individual sequencing results as raw and clean paired-end reads, mapped reads and mapping rates for the two spatial tactics of the focal 10 cheetah males (10 IDs), each sampled as floater (F) and again as territory holder (T).

| **ID** | **Sample ID** | **Raw paired-end reads** | **Clean paired-end reads** | **Mapped reads** | **Mapping rate [%]** |
| --- | --- | --- | --- | --- | --- |
| ID1 | F1 | 49,437,682 | 24,555,170 | 22,723,354 | 92.54 |
| ID1 | T1 | 63,454,218 | 31,542,968 | 28,480,145 | 90.29 |
| ID2 | F2 | 46,175,782 | 22,930,672 | 20,477,090 | 89.30 |
| ID2 | T2 | 54,957,562 | 27,203,340 | 24,526,531 | 90.16 |
| ID3 | F3 | 61,951,426 | 30,860,281 | 29,878,924 | 96.82 |
| ID3 | T3 | 44,158,400 | 21,946,390 | 20,085,336 | 91.52 |
| ID4 | F4 | 48,161,006 | 23,874,267 | 23,363,357 | 97.86 |
| ID4 | T4 | 65,024,276 | 32,293,213 | 29,367,447 | 90.94 |
| ID5 | F5 | 58,473,896 | 28,938,655 | 26,120,030 | 90.26 |
| ID5 | T5 | 65,199,362 | 32,338,449 | 31,044,911 | 96.00 |
| ID6 | F6 | 52,893,832 | 26,369,693 | 24,075,529 | 91.30 |
| ID6 | T6 | 60,700,776 | 30,227,217 | 23,281,002 | 77.02 |
| ID7 | F7 | 64,284,028 | 32,044,731 | 31,150,683 | 97.21 |
| ID7 | T7 | 64,453,734 | 32,133,384 | 28,354,498 | 88.24 |
| ID8 | F8 | 46,537,746 | 23,218,166 | 22,119,946 | 95.27 |
| ID8 | T8 | 55,581,122 | 27,722,698 | 26,070,425 | 94.04 |
| ID9 | F9 | 101,453,882 | 50,617,721 | 49,463,636 | 97.72 |
| ID9 | T9 | 65,090,378 | 32,447,653 | 30,383,982 | 93.64 |
| ID10 | F10 | 53,332,672 | 26,601,088 | 26,095,667 | 98.10 |
| ID10 | T10 | 55,278,850 | 27,560,076 | 26,978,558 | 97.89 |

**Table S3:** Predictive performance and coherence of biomarker models for classifying male cheetah spatial tactic.

| **Method** | **Accuracy (range)** | **Precision (range)** | **Sensitivity (range)** | **Specificity (range)** | **F-measure (range)** |
| --- | --- | --- | --- | --- | --- |
| PCa + ElasticNet | 0.76  (0.70–0.85) | 0.74  (0.67–0.82) | 0.81  (0.70–0.90) | 0.71  (0.60–0.80) | 0.77  (0.70–0.86) |
| PCo + ElasticNet | 0.74  (0.65–0.80) | 0.72  (0.64–0.75) | 0.78  (0.60–0.90) | 0.69  (0.60–0.70) | 0.75  (0.63–0.82) |
| nP + ElasticNet | 0.78  (0.75–0.85) | 0.74  (0.71–0.82) | 0.86  (0.80–0.90) | 0.71  (0.70–0.80) | 0.80  (0.76–0.86) |
| random_PCa | 0.65  (0.50–0.80) | 0.63  (0.50–0.78) | 0.69  (0.50–0.90) | 0.60  (0.40–0.80) | 0.66  (0.50–0.80) |
| random_PCo | 0.65  (0.50–0.80) | 0.64  (0.50–0.78) | 0.69  (0.40–0.90) | 0.61  (0.50–0.80) | 0.66  (0.44–0.80) |
| random_nP | 0.66  (0.50–0.80) | 0.65  (0.50–0.78) | 0.71  (0.50–0.90) | 0.61  (0.40–0.80) | 0.67  (0.49–0.82) |

*Performance of Random Forest models built using biomarkers derived from three analytical methods (PCa, PCo and nP) and selected by ElasticNet. Metrics (Accuracy, Precision, Sensitivity, Specificity, F-measure) were calculated on held-out individuals from 10-fold cross-validation and averaged over 100 repetitions. Accuracy represents the overall proportion of correctly classified individuals. Precision indicates the proportion of predicted floaters that were indeed floaters. Sensitivity denotes the proportion of correctly identified floater males and specificity that of territorial males. The F-measure represents the harmonic mean of Precision and Sensitivity, providing a balanced indicator of classification performance.*

**Table S4:** Putative epigenetic biomarkers for discriminating floaters from territorial male spatial tactic, including Chromosome, Start, End, GeneID/Intergenic, and ElasticNet coefficient ranges for each method (PCa, PCo, nP).

*Positive coefficient values are hypermethylated DMRs in territorial males, negative coefficient values (-) are hypomethylated DMRs in territorial males.* *ElasticNet parameters indicate effect size and whether DMRs were detected by nP or PCo and PCa analyses, or several. Most DMRs were hypermethylated in territorial males, with varying effect size. None of the DMRs and genes identified as biomarkers overlapped with the ones found in the age class comparison between younger and older adult floaters. This panel has the potential for using a small set of epigenetic markers to accurately determine male cheetah spatial tactic-shift from a random biological sample.*

**References**

1.	Putri, G. H., Anders, S., Pyl, P. T., Pimanda, J. E. & Zanini, F. Analysing high-throughput sequencing data in Python with HTSeq 2.0. *Bioinformatics* **38**, 2943–2945 (2022).

2.	Steegen, S., Tuerlinckx, F., Gelman, A. & Vanpaemel, W. Increasing Transparency Through a Multiverse Analysis. *Perspect Psychol Sci* **11**, 702–712 (2016).
